# Serum calprotectin: a promising biomarker in rheumatoid arthritis and axial spondyloarthritis

**DOI:** 10.1186/s13075-020-02190-3

**Published:** 2020-05-06

**Authors:** Matthias Jarlborg, Delphine S. Courvoisier, Céline Lamacchia, Laura Martinez Prat, Michael Mahler, Chelsea Bentow, Axel Finckh, Cem Gabay, Michael J. Nissen, on behalf of the physicians of the Swiss Clinical Quality Management (SCQM) registry

**Affiliations:** 1 Geneva University Hospital, 26 avenue de Beau-Séjour, 1206 Geneva, Switzerland; 2 Inova Diagnostics, Inc, San Diego, USA

**Keywords:** Serum calprotectin, S100A8/S100A9, Biomarker, Rheumatoid arthritis, Psoriatic arthritis, Axial spondyloarthritis

## Abstract

**Background:**

Calprotectin (S100A8/S100A9 protein) is known as a damage-associated molecular pattern (DAMP) protein and reflects mainly neutrophil activation. Serum calprotectin levels might be a good alternative to acute-phase protein as a biomarker in inflammatory rheumatic diseases. The aim of this study is to investigate the association of serum calprotectin with disease activity and severity in rheumatoid arthritis (RA), axial spondyloarthritis (axSpA), and psoriatic arthritis (PsA).

**Methods:**

Serum calprotectin was measured in patients with RA, axSpA, and PsA from the prospective Swiss Clinical Quality Management (SCQM) registry. Asymptomatic first-degree relatives of RA patients were used as healthy controls (HC). Outcomes included swollen joint count (SJC), Disease Activity Score (DAS), Health Assessment questionnaire (HAQ), joint radiographs, and ultrasound power Doppler (USPD) score for RA; Bath Ankylosing Spondylitis Disease Activity Index (BASDAI), Ankylosing Spondylitis Disease Activity Score (ASDAS) and coxitis for axSpA; and SJC and Disease Activity Index for PSoriatic Arthritis (DAPSA) for PsA. Comparison of outcomes by calprotectin quartile levels was performed using Kruskal-Wallis tests for continuous outcomes or trend tests for categorical outcomes.

**Results:**

A total of 1729 subjects [RA = 969, axSpA = 451, PsA = 237, and HC = 72] were included. Median levels of serum calprotectin were higher in each disease group compared to HC (*p* < 0.01). In RA patients, all clinical outcomes were statistically different between quartiles of serum calprotectin, indicating an association between calprotectin levels and higher disease activity (SJC, DAS, and USPD scores) and severity (joint radiographs and HAQ). In axSpA, an association between calprotectin levels and ASDAS score (*p* < 0.01) and prevalence of coxitis (*p* = 0.02) was observed. For PsA patients, SJC and DAPSA did not differ across calprotectin quartiles.

**Conclusions:**

This large study supports the association of serum calprotectin levels with disease activity in both RA and axSpA, but not in PsA.

## Background

Serum biomarkers are frequently used as diagnostic tools as well as for evaluation of disease activity and treatment response in inflammatory rheumatic diseases (IRD). C-reactive protein (CRP) and erythrocyte sedimentation rate (ESR) are routinely measured for disease monitoring. However, less than 50% of patients with axial spondyloarthritis (axSpA) and psoriatic arthritis (PsA) present with an elevated CRP [1, 2]. Furthermore, anti-interleukin (IL)-6 therapies (such as tocilizumab) have a direct blocking effect on CRP [3, 4], which precludes its use to assess treatment response or the occurrence of infection. Thus, new biomarkers are required, particularly in rheumatoid arthritis (RA), axSpA, and psoriatic arthritis (PsA).

Calprotectin (or leukocyte protein L1) is a potentially interesting biomarker for a number of IRD. Calprotectin is a heterodimeric complex of two non-covalently associated calcium-binding proteins, S100A8 and S100A9. They belong to the S100 proteins family which regroups 25 members [5]. These two proteins are also known as myeloid-related protein (MRP) 8 and 14 or calgranulin A and B. Although debated, it seems that they are functionally active when present in their heterodimeric form [6], forming the calprotectin complex (S100A8/S100A9). Calprotectin is stored in large amounts in the granulocyte cytosol (40–60% of cytosolic protein content) and has both intracellular and extracellular functions. Inside the cells, it regulates calcium homeostasis, interacts with the cytoskeleton and microtubules and plays a role in intracellular trafficking of phagocytes. Its role for leukocyte transmigration has been recently shown in a mouse model [7]. When released, calprotectin functions as a damage-associated pattern molecules (DAMP) or alarmin, promoting the inflammatory response.

The normal serum levels of calprotectin are estimated to range between 0.1 and 1.6 μg/ml and can be elevated in numerous conditions such as infection, inflammation, or cancer [8]. More recently, a cut-off above 0.9 μg/ml was proposed to distinguish RA from non-inflammatory arthritis [9]. Given its low molecular weight (36.5 kDa), calprotectin may diffuse from inflamed tissues to the blood circulation. Indeed, serum concentrations seem to reflect synovial concentrations, with a ratio of 1:2–3 according to some studies [10, 11]. Both synovial and serum calprotectin levels are elevated in RA, but not in osteoarthritis [10]. A recent meta-analysis demonstrated that circulating and synovial calprotectin correlates with disease activity in RA [12]. Calprotectin levels also correlated with ultrasound synovitis, particularly with power Doppler signal which was not the case for either CRP or ESR [13–15]. Furthermore, calprotectin is potentially an independent predictor of radiological progression [14, 15]. Inconsistent results have been reported regarding the use of calprotectin as a predictor of treatment response [16–19]. Very little is known about calprotectin and IL-6 inhibition therapies. A single cross-sectional study investigated calprotectin and IL-6 inhibitors. In this cohort of 33 RA patients receiving tocilizumab, serum calprotectin levels seemed to be an accurate biomarker for assessing disease activity [20]. Serum calprotectin levels are also significantly elevated in axSpA and non-radiographic (nr)-axSpA and correlate with CRP as well as with clinical (BASDAI, ASDAS) and radiological (SPARCC) disease activity scores [21, 22]. Moreover, some authors reported that calprotectin was predictive for progression of structural damage in the spine of patients with axSpA [23, 24]. Among patients with PsA, serum calprotectin levels also correlated with disease activity, with higher levels in patients with symmetrical polyarthritis compared to patients with a mono- or oligo-articular presentations [25]. A recent study could also confirm this correlation in early PsA, as well as in early RA [26]. In psoriasis, serum calprotectin levels correlate with the Psoriasis Area and Severity Index (PASI) score [27].

The overall aim of the study was to compare the value of serum calprotectin as a biomarker for disease activity and severity in RA, axSpA, and PsA, in a cohort of patients from the Swiss Clinical Quality Management (SCQM) registry.

## Methods

### Design and study population

This study is a nested case-control study within the Swiss Clinical Quality Management (SCQM) registry. This national cohort was established in 1996 and includes patients with a confirmed diagnosis of RA, axSpA, and PsA. The diagnosis is established according to the expertise of board-certified rheumatologists. Clinicians participating in the SCQM are office or hospital-based rheumatologists. They provide clinical patient data and ultrasound examination data (for RA patients), on a regular basis. In addition, patients fill out a number of patient-reported outcome (PRO) questionnaires at each visit. This registry has been described in more detail in a previous article [28].

The present study includes all participants in the SCQM registry with a blood sample available between March 2011 and April 2013. Asymptomatic first-degree relatives (FDRs) of RA patients, from the SCREEM-RA cohort [29], were used as healthy controls (HC). Participants from this Swiss multicenter cohort study of RA FDRs were matched to the RA population in terms of age and sex. They had no signs of autoimmunity, defined as the absence of anti-citrullinated peptide antibodies (ACPAs), rheumatoid factor (RF), and without shared epitope and no joint complaints. This study was approved by the Ethics Committee of the University Hospital of Geneva, and all individuals signed an informed consent form prior to enrolment, in accordance with the Declaration of Helsinki.

### Exposure of interest and outcome parameters

Serum calprotectin levels were measured using the QUANTA Lite Calprotectin ELISA (Inova Diagnostics, San Diego, Research Use Only for serum/plasma). All values are expressed in micrograms per milliliter. Levels of CRP (mg/l) and ESR (mm/h) were also documented in each disease group. Differences in calprotectin levels in each disease group were compared with the Wilcoxon test. As the distribution of serum calprotectin in our cohort is non-normal (Shapiro-Wilk normality test *w* = 0.815, *p* value < 0.001) and as the relationships between calprotectin and the outcome parameters are non-linear, we chose to categorize calprotectin levels into quartiles for each disease group. Comparison of clinical outcomes by calprotectin quartile levels was then performed using the Kruskal-Wallis tests for continuous outcomes or trend tests for categorical outcomes.

We examined the cut-off for the serum calprotectin level as marker for disease activity with a receiver operating characteristic (ROC) analysis.

For RA patients, outcome measures included clinically assessed scores such as the swollen joint count (SJC), tender joint count (TJC), and self-reported scores such as the Rheumatoid Arthritis Disease Activity Index (RADAI) and the health assessment questionnaire (HAQ) disability index. We also used composite scores such as the Clinical Disease Activity Index (CDAI) and Disease Activity Score (DAS28). Hand and feet radiographs were assessed regularly over time (until 2016) with a validated scoring method, the Ratingen score [30]. Radiographs are evaluated prospectively by an assessor blinded to the clinical information. Multivariable analyses were corrected for age, sex, smoking status, disease duration, disease activity (DAS28), number of prior biologics, and calendar year of biosampling.

For musculoskeletal ultrasound assessments in RA patients, we used a standardized semiquantitative (0 to 3) scoring system for grayscale (GS) mode and Power Doppler (PD). This score was developed by the Swiss Sonography in Arthritis and Rheumatism (SONAR) group, based on the recommendations from the OMERACT group [31], and has demonstrated good correlation with clinical disease activity and sensitivity to change in an observational cohort study [32]. The SONAR score includes 22 joints (the same joints as the DAS28, but excluding the 2 joints in the thumbs and the shoulders). A total GS-mode score of at least 10 (out of 66) or a total PD-score of at least 1 (out of 66) was defined as a positive SONAR score in this study, suggesting active inflammatory disease. Multivariable regression models were used to compare the association of CRP and calprotectin with USPD, and the proportions of explained variance were estimated using *R*^2^.

In a subgroup analysis, we performed the ROC and Kruskal-Wallis analyses among patients treated with tocilizumab during at least 1 month (*n* = 92) to compare the capacity of calprotectin and CRP to detect a DAS28-ESR score equal or superior to 3.2 (corresponding to moderate or severe disease activity). We restricted the analysis to the subjects who had provided a blood sample within 30 days of clinical examination.

For axSpA patients, our main outcomes were clinical disease activity scores focusing on axial involvement and disability, such as the Bath Ankylosing Spondylitis Disease Activity Index (BASDAI), the Bath Ankylosing Spondylitis Functional Index (BASFI), and the Ankylosing Spondylitis Disease Activity Score (ASDAS) [33]. We also examined the physician global disease activity scale (graded from 0 to 10). Clinical signs of peripheral disease were also investigated, such as the SJC, coxitis, enthesitis, and dactylitis.

In PsA, the TJC, the SJC, and the Disease Activity Index for PSoriatic Arthritis (DAPSA) were the main outcomes for articular involvement. For skin evaluation, we dichotomized the reported extent of skin involvement into absent (no lesion and almost no lesion) or present (mild, moderate or severe involvement). The Dermatology Life Quality Index (DLQI) self-reported score was also included.

All analyses were performed using R v3.5.1 (R foundation, Vienna).

## Results

### Population characteristics

A total of 1729 subjects [RA = 969, axSpA = 451, PsA = 237, and HC = 72] were included in the study. Clinical characteristics of the different populations are shown in Table 1. Among RA patients, 751 (77.5%) met the 2010 ACR/EULAR classification score. In axSpA, 361 (80.0%) were positive for the Assessment of Spondyloarthritis International Society (ASAS) or the Modified New-York Criteria. In PsA, 198 (83.5%) had positive Classification Criteria for Psoriatic Arthritis (CASPAR). The median time interval between blood collection and clinical examination (outcomes) was 0 days (IQR 0 to 6 days). Musculoskeletal ultrasound evaluations were available in 209 RA patients. The median time interval between the blood collection and the date of ultrasound assessment was 0 days (IQR − 1.5 to 22.5). Among them, 85 (40.7%) had ultrasound and blood collection performed on the same day. A total of 851 RA patients (87.8%) had at least one available joint radiograph, with a median Ratingen score of 4 (range 1–17). RA patients and the matched HC group demonstrated a higher percentage of women and older age than patients with axSpA and PsA. There were no differences in CRP (*p* = 0.39) or ESR (*p* = 0.62) levels between the 3 disease groups.

**Table 1:** Population characteristics and median serum calprotectin levels in each group. All results are mean (with standard deviation), except those with an asterisk* which are median [with interquartile range] and those marked with a “^§^” which are number of patients (%). *Abbreviations*: *BMI* body mass index, *CRP* C-reactive protein, *ESR* erythrocyte sedimentation rate, *GCS* glucocorticoids, *cDMARDS* conventional disease-modifying anti-rheumatic drugs, *bDMARDS* biological disease-modifying anti-rheumatic drugs, *TNFi* TNF inhibitors

	Rheumatoid arthritis	Axial spondyloarthritis	Psoriatic arthritis	Healthy control	*p* value
*n*	969	451	237	72	
Gender = male^§^	250 (25.8)	268 (59.4)	136 (57.4)	13 (18.1)	< 0.01
Age, year	57.6 (13.2)	44.0 (12.3)	51.5 (12.2)	56.5 (8.3)	< 0.01
Disease duration, year	10.9 (10.1)	9.9 (9.8)	7.9 (8.4)	–	< 0.01
BMI kg/m^2^	26.2 (5.4)	26.0 (4.9)	27.5 (5.4)	24.1 (3.4)	< 0.01
Smoker^§^	435/758 (57.4)	146/264 (55.3)	101/168 (60.1)	34/72 (47.2)	0.37
Calprotectin μg/ml*	2.6 [1.7, 4.3]	2.4 [1.6, 3.9]	2.2 [1.4, 3.4]	1.2 [0.8, 2.0]	< 0.01
CRP mg/l*	3.0 [1.0, 7.9]	3.0 [1.0, 8.0]	3.0 [1.0, 7.9]	–	0.39
ESR mm/h*	12.0 [6.0, 22.0]	8.0 [4.0, 17.0]	8.0 [3.0, 14.0]	–	0.62
Swollen joint count*	1.0 [0.0, 3.0]	0.0 [0.0, 0.0]	0.0 [0.0, 2.0]	–	< 0.01
Disease activity score^1^	2.8 [2.0, 3.9]	2.3 [1.5, 3.2]	12 [5.1, 24.0]		–
Patients on GCS^§^	277 (28.6)	3 (0.7)	19 (8.0)	–	< 0.01
Patients on cDMARDS^§^	645 (66.6)	75 (16.6)	142 (59.9)	–	< 0.01
Patients on bDMARDS^§^	557 (57.5)	184 (40.8)	108 (45.6)	–	< 0.01
TNFi^§^	205 (21.2)	180 (39.9)	100 (42.2)		< 0.01
Tocilizumab^§^	92 (9.5)	3 (0.7)	3 (1.3)		< 0.01
Rituximab^§^	172 (17.8)	0 (0.0)	1 (0.4)		< 0.01

^1^Composite scores: DAS28-ESR for RA, ASDAS-CRP for AxSpA, and DAPSA for PsA are presented

**Fig. 1:**
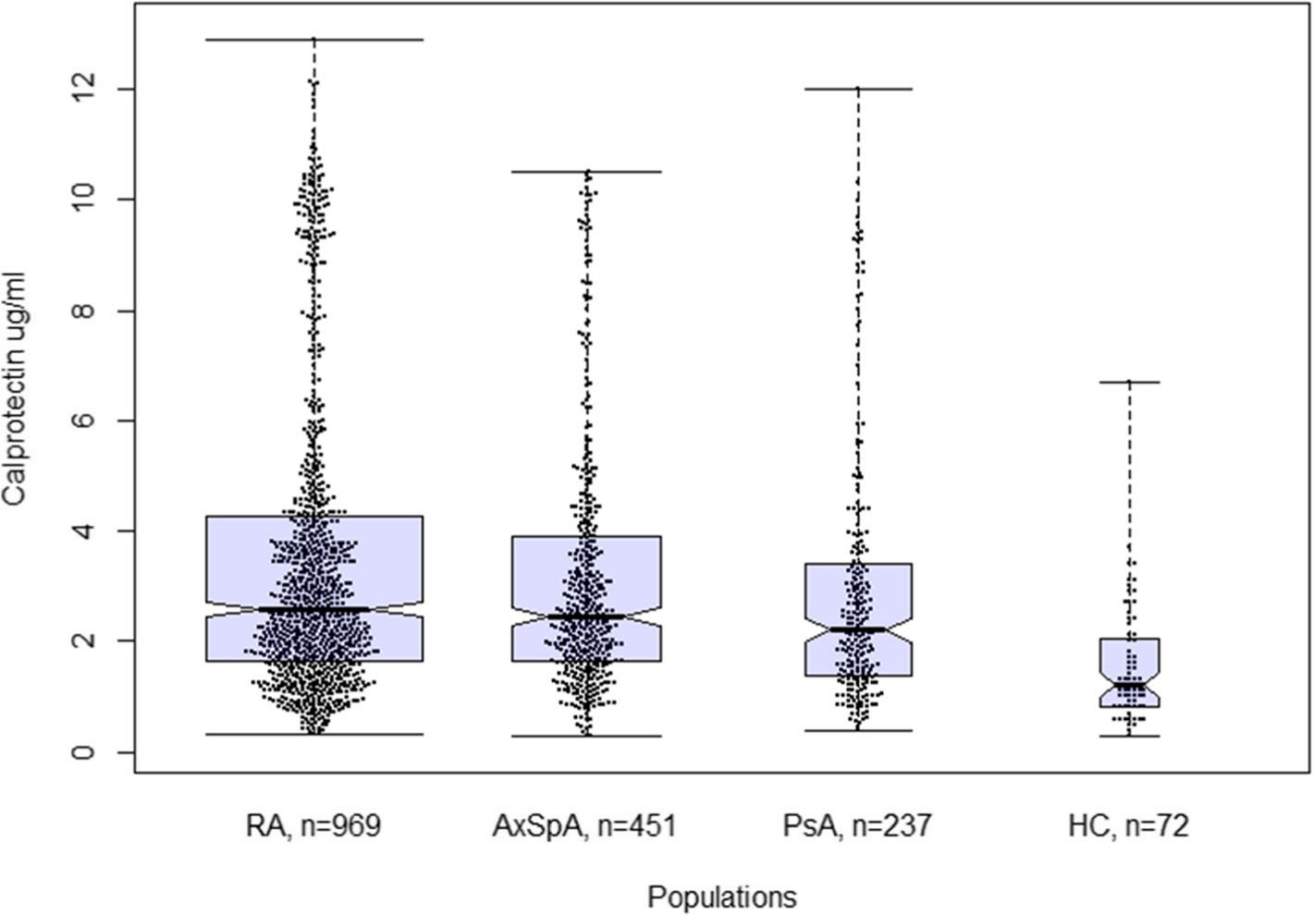
Boxplots of median and interquartile range of serum calprotectin level by diagnosis. The width of the boxplot reflects the size of the group. Abbreviations: RA, rheumatoid arthritis; AxSpA, axial spondyloarthritis; PsA, psoriatic arthritis; HC, healthy control

**Fig. 2:**
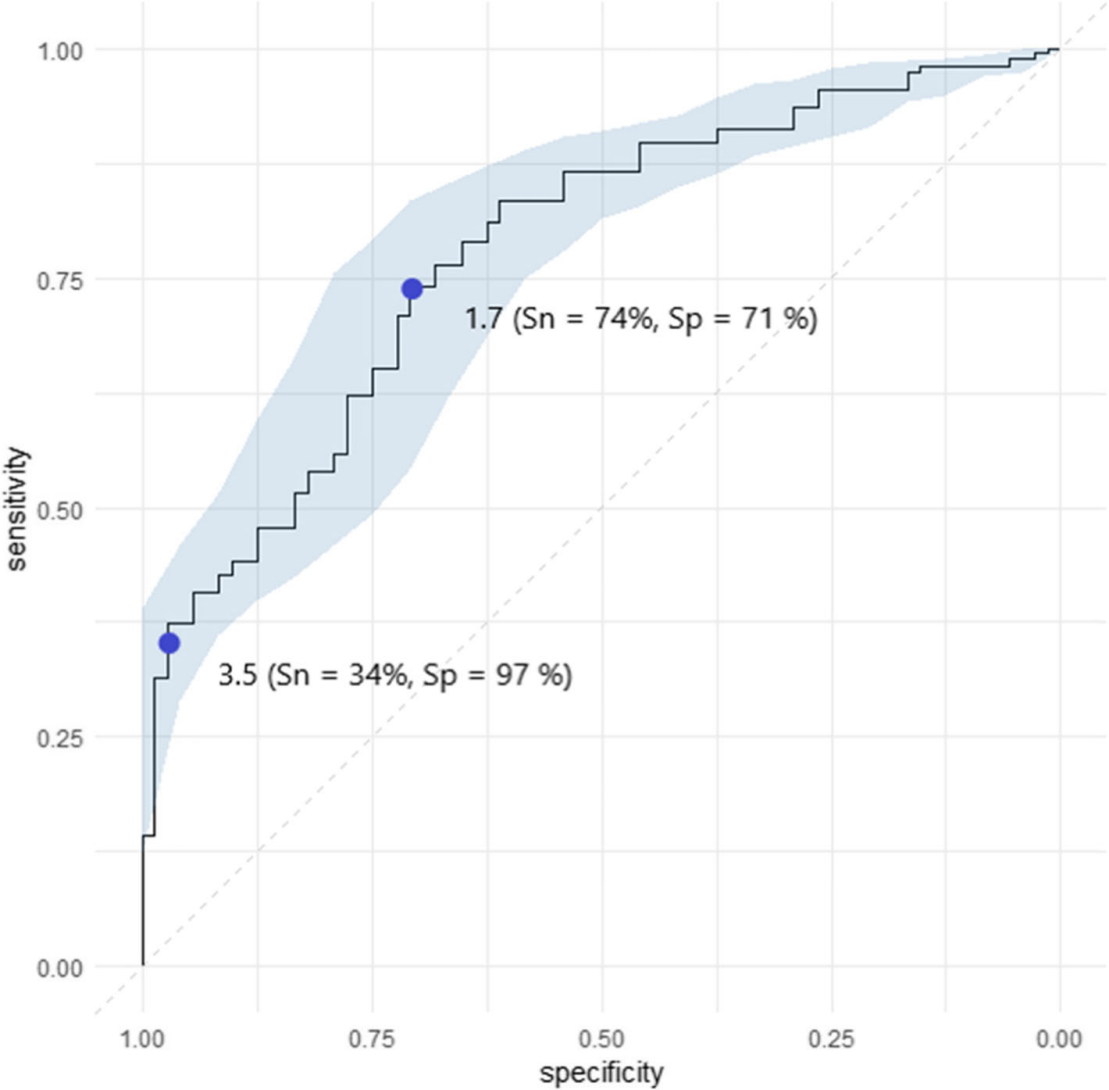
Receiver operating characteristic (ROC) curve of serum calprotectin as diagnostic marker in rheumatoid arthritis. Rheumatoid arthritis (*n* = 969) versus healthy individuals (*n* = 72). Thresholds of 3.5 μg/ml and 1.7 μg/ml (threshold with the highest sum sensitivity + specificity) and confidence interval for sensitivity are plotted. Area under the curve: 0.78, 95% CI 0.73–0.83 (DeLong). Abbreviations: Sn, sensitivity; Sp, specificity

Median levels of serum calprotectin for each of the 3 IRD groups were significantly higher compared to the HC (*p* < 0.01; Fig. 1). No significant difference in calprotectin levels between RA patients and axSpA patients was observed (*p* = 0.19). The highest calprotectin levels were found in RA patients (median 2.6 [1.7–4.3] μg/ml; mean 3.5 μg/ml ± SD 2.7) and the lowest in the HC group (median 1.2 [0.8, 2.0] μg/ml; mean 1.5 μg/ml ± SD 1.0). There were no differences in median calprotectin levels between young onset RA (*n* = 771) and elderly onset RA (*n* = 177), with the cut-off at 60 years of age (*p* = 0.59). A ROC analysis demonstrated that serum calprotectin could discriminate RA patients from matched HC with a good specificity but poor sensitivity (Fig. 2). A calprotectin threshold of 3.5 μg/ml (corresponding to 2 standard deviations above the mean level in HC) yielded 97% specificity and 34% sensitivity.

### Serum calprotectin as a marker of disease activity in rheumatoid arthritis

In RA, higher calprotectin serum levels were associated with more severe forms of the disease. The proportion of males was significantly higher towards the upper quartile (*p* = 0.01). No differences existed with regard to age, body mass index, or disease duration between quartiles. All clinical disease activity scores were statistically different across serum calprotectin quartiles, showing higher disease activity with the highest serum calprotectin level quartile (Table 2). This was true for PRO scores (RADAI, HAQ), clinical assessments (TJC, SJC), and composite scores (DAS28, CDAI). In supplementary figure S1, a ROC analysis shows the performance of serum calprotectin to discriminate low from moderate/high disease activity according to DAS28-ESR in a population of early RA (defined by a disease duration of less than 24 months). The calprotectin threshold of 3.5 μg/ml yielded 79% specificity and 57% sensitivity.

**Table 2:** Characteristics of the patients with rheumatoid arthritis stratified by calprotectin percentiles. All results are mean (with standard deviation), except those with an asterisk* which are median [with interquartile range] and those marked with a “^§^” which are number of patients (%). *Abbreviations*: *BMI* body mass index, *CRP* C-reactive protein, *ESR* erythrocyte sedimentation rate, *RADAI* Rheumatoid Arthritis Disease Activity Index, *HAQ* Health Assessment Questionnaire, *CDAI* Clinical Disease Activity Index, *DAS28* 28-joints Disease Activity Score, *US* ultrasound, *SONAR* Swiss Sonography in Arthritis and Rheumatism, *ACR/EULAR* American College of Rheumatology/European League Against Rheumatism, *RF* rheumatoid factor, *ACPA* anti-citrullinated peptide antibodies

Serum calprotectin quartile [μg/ml]	0–1.7	1.7–2.6	2.6–4.3	4.3–13	*p* value
*n*	243	242	242	242	
Age years	56.6 (13.0)	57.2 (13.0)	58.3 (13.8)	58.3 (12.8)	0.41
Elderly onset^§1^	38 (15.6)	51 (21.1)	45 (18.6)	43 (17.8)	0.77
Disease duration, years	10.6 (10.1)	10.8 (10.0)	10.8 (10.0)	11.3 (10.5)	0.92
Gender = male^§^	53 (21.8)	56 (21.3)	65 (26.9)	76 (31.4)	0.01
BMI kg/m^2^	25.3 (5.0)	26.3 (5.5)	26.3 (5.9)	27.0 (5.2)	0.16
CRP mg/l*	2.0 [1.0, 4.6]	2.0 [1.0, 7.0]	2.2 [1.0, 7.0]	4.2 [2.0, 12.0]	< 0.01
ESR mm/h*	8.0 [5.0, 16.0]	10.0 [6.0, 20.0]	12.0 [5.5, 22.5]	16.0 [8.0, 30.0]	< 0.01
Tender joint count	2.6 (4.3)	3.2 (4.8)	3.6 (5.2)	4.0 (5.6)	0.02
Swollen joint count	1.3 (2.5)	1.7 (2.8)	2.2 (3.3)	3.4 (4.5)	< 0.01
RADAI score	2.3 (1.8)	2.9 (2.2)	2.9 (2.2)	3.3 (2.2)	< 0.01
HAQ	0.6 (0.6)	0.8 (0.7)	0.8 (0.7)	1.0 (0.7)	< 0.01
CDAI	7.3 (7.9)	9.8 (9.9)	11.2 (11.7)	12.3 (13.4)	< 0.01
DAS28 CRP	2.5 (1.0)	2.8 (1.1)	2.8 (1.2)	3.2 (1.4)	< 0.01
DAS28 ESR	2.6 (1.3)	2.9 (1.3)	3.0 (1.3)	3.5 (1.5)	< 0.01
DAS28 ESR moderate/high^§2^	58/190 (30.5)	78/196 (39.8)	83/193 (43.09)	118/200 (59.0)	< 0.01
DAS28 ESR remission ^§3^	103/190 (54.2)	86/196 (43.9)	78/193 (40.4)	60/200 (30.0)	< 0.01
US score B mode score	8.9 (6.5)	9.1 (6.3)	10.4 (5.3)	11.6 (8.7)	0.13
US Power Doppler score	2.2 (5.9)	2.0 (4.0)	2.0 (3.0)	4.7 (6.1)	0.01
SONAR positive^§^	28/44 (63.6)	30/47 (63.8)	31/47 (66.0)	58/71 (81.7)	0.02
ACR/EULAR 2010 Criteria	6.7 (2.1)	7.1 (2.1)	7.3 (2.2)	7.6 (2.1)	< 0.01
RF positive^§^	157/232 (67.7)	164/233 (70.4)	172/228 (75.4)	179/236 (75.8)	0.02
ACPA positive^§^	144/226 (63.7)	145/223 (65.0)	158/217 (72.8)	159/227 (70.0)	0.06
Rheumatoid nodules^§^	36/232 (15.5)	51/232 (21.9)	46/226 (20.3)	59/223 (26.4)	0.01

^1^Elderly onset = age of onset after 60 years of age

^2^Proportion of patients with a DAS28 ESR ≥ 3.2

^3^Proportion of patients with a DAS28 ESR < 2.6

A total of 560 RA patients (57.8%) had a normal CRP level at the sampling date, defined as less than 5 mg/l. In this subgroup, without acute phase reactants, we also demonstrated a statistically significant association with calprotectin quartiles levels and SJC (*p* = 0.02). The median calprotectin levels were also statistically different between patients with moderate/high disease activity (DAS28-ESR ≥ 3.2, *n* = 165) and low disease activity (DAS28-ESR < 3.2, *n* = 352) in this subgroup, with a median [IQR] of respectively 2.8 [1.7–4.3] μg/ml and 2.2 [1.5–3.6] μg/ml (Wilcoxon test, *p* = 0.005).

Regarding the subgroup of 209 patients with available ultrasound examinations, USPD scores were significantly higher in the uppermost quartile (*p* = 0.007). In univariable regression models, both CRP (*R*^2^ = 0.02, *p* = 0.04) and calprotectin (*R*^2^ = 0.10, *p* < 0.001) were associated with elevated USPD scores, albeit with a very low proportion of variance explained. Multivariable model analysis revealed that calprotectin alone was independently associated with USPD scores. The addition of CRP to this model did not improve the association (likelihood ratio test between models *p* = 0.14). Furthermore, the combination of calprotectin and CRP was more predictive than CRP alone (*p* < 0.001). The number of patients with a positive SONAR score also differed significantly difference across quartiles (*p* = 0.02). Supplementary figure S2 demonstrates the sensitivity and specificity of calprotectin and CRP to detect a positive SONAR score, suggestive of active disease. The ability of calprotectin to detect a positive SONAR score was better in patients with a disease duration of less than 5 years.

Among the 92 (9.5%) RA patients treated with tocilizumab, 56 (60.9%) had an available blood collection within 30 days of the clinical examination. The median levels of serum calprotectin were statistically higher in patients with one or more swollen joints compared to patients with no swollen joints (*p* = 0.03; supplementary figure S3). We found no difference for CRP levels (*p* = 0.1; supplementary figure S3). According to the DAS28-ESR, 12 of 49 subjects with data available (24.5%) had moderate or high disease activity (score ≥ 3.2). The mean calprotectin level differed significantly between the group in remission or low disease activity (3.2 μg/ml ± 2.6 SD)) and the group with moderate to high disease activity (6.5 μg/ml ± 4.2 SD) (*p* = 0.002). This was not the case for the median level of CRP (*p* = 0.17). Median level of calprotectin and CRP are described in supplementary figure S4. The ROC analysis on the same figure demonstrates that calprotectin is more accurate to detect patients with a DAS28-ESR score above 3.2 than CRP, with an area under the curve of 0.8 and 0.71 respectively.

### Serum calprotectin as a marker of disease severity in rheumatoid arthritis

Higher quartiles of serum calprotectin were associated with a significantly higher prevalence of RF seropositivity (*p* = 0.02) and a significantly higher number of items of the ACR/EULAR classification score (*p* < 0.01). The presence of rheumatoid nodules was also more frequently reported in the highest quartile of calprotectin (*p* = 0.04). Similar results were obtained with the subgroup of patients known to present positive 2010 ACR/EULAR classification criteria.

A total of 70 RA patients (7.2%) with the highest calprotectin levels (above 9.46 μg/ml) had significantly higher baseline Ratingen scores compared to patients with normal calprotectin levels, in both univariable and multivariable analyses (*p* = 0.04 and *p* = 0.02, respectively). Supplementary figure S5 demonstrates the evolution of the Ratingen score over time in relation to the calprotectin quartile levels. The highest quartile was associated with a significantly higher baseline Ratingen score (*p* = 0.01), although the change over time was similar across quartiles. These results remained significant in adjusted analyses (*p* = 0.01).

### Serum calprotectin as a marker of disease activity in axial spondyloarthritis

In axSpA, the mean calprotectin level was 3.2 μg/ml (SD ± 2.7). As shown in Table 3, an association between higher calprotectin quartiles and higher ASDAS scores (*p* = 0.01) was observed. When each criterion of ASDAS score was tested separately, only CRP was different across quartiles. Physician global disease activity scale was also significantly higher across quartiles (*p* = 0.02). Although there was an association between calprotectin quartiles and the proportion of patients with moderate or high disease activity according to ASDAS score (*p* = 0.04), we did not find any association with the proportion of patients in remission. When focusing on the extra-axial manifestations, we found no association between serum calprotectin quartiles and clinical manifestations, including SJC, enthesitis, or dactylitis. However, there was significantly more inflammatory hip involvement in the highest calprotectin quartile (*p* = 0.02). With the exception of a higher proportion of males across the quartiles, there were no differences regarding the other population characteristics (age, body mass index or disease duration). Same results were obtained with the subgroup meeting the ASAS or the Modified New-York criteria.

**Table 3:** Characteristics of the patients with axial spondyloarthritis stratified by calprotectin percentiles. All results are mean (with standard deviation), except those with an asterisk* which are median [with interquartile range], and those marked with a “^§^” which are number of patients (%). *Abbreviations*: *BMI* body mass index, *CRP* C-reactive protein, *ESR* erythrocyte sedimentation rate, *BASDAI* Bath Ankylosing Spondylitis Disease Activity Index, *BASFI* Bath Ankylosing Spondylitis Functional Index, *ASDAS* Ankylosing Spondylitis Disease Activity Score

Serum calprotectin quartile [μg/ml]	0–1.6	1.6–2.4	2.4–3.9	3.9–13	*p* value
*n*	113	113	112	113	
Disease duration, years	10.4 (10.2)	8.5 (9.8)	9.9 (9.8)	10.6 (9.4)	0.37
Age years	45.9 (11.4)	44.4 (12.7)	43.0 (11.3)	42.7 (13.7)	0.20
Gender = male^§^	62 (54.9)	62 (54.9)	67 (59.8)	77 (68.1)	0.03
BMI kg/m^2^	25.3 (4.7)	26.3 (4.8)	26.3 (5.4)	26.3 (4.7)	0.44
ESR mm/h*	6.0 [4.0, 11.5]	8.0 [4.0, 17.5]	8.0 [4.0, 16.0]	10.0 [4.0, 25.0]	0.01
CRP mg/l*	3.0 [1.0, 8.0]	2.0 [1.0, 8.0]	3.0 [1.0, 8.0]	4.0 [2.0, 13.0]	< 0.01
BASDAI score	3.6 (2.3)	4.0 (2.4)	3.5 (2.4)	4.1 (2.3)	0.19
BASFI score	2.1 (2.3)	2.7 (2.6)	2.3 (2.3)	2.8 (2.5)	0.22
ASDAS score	2.1 (0.9)	2.4 (1.0)	2.2 (1.1)	2.7 (1.0)	0.01
ASDAS moderate/high^§1^	40/79 (50.6)	47/81 (58.0)	37/65 (56.9)	49/72 (68.1)	0.04
ASDAS remission^§2^	14/79 (17.7)	16/81 (19.8)	20/65 (30.8)	4/72 (5.56)	0.20
Physician global disease activity	1.9 (1.6)	2.4 (2.0)	2.2 (2.0)	2.6 (1.8)	0.02
Swollen joint count	0.4 (0.9)	0.6 (1.8)	0.4 (0.9)	0.3 (1.2)	0.46
Hip involvement^§^	3 (2.7)	3 (2.7)	5 (4.5)	10 (8.8)	0.02
Enthesitis^§^	32 (28.3)	38 (33.6)	40 (35.7)	36 (31.9)	0.68
Dactylitis^§^	2 (1.8)	0 (0.0)	5 (4.5)	3 (2.7)	0.51

^1^Proportion of patients with an ASDAS score ≥ 2.1

^2^Proportion of patients with an ASDAS score < 1.3

### Serum calprotectin as a marker of disease activity in psoriatic arthritis

PsA patients had significantly lower calprotectin levels (mean 2.9 μg/ml ± 2.3 SD) compared to RA (*p* < 0.01) and axSpA (*p* = 0.02) patients. All clinical activity scores (TJC, SJC, and DAPSA) did not differ across calprotectin quartiles (Table 4). For the other biological markers, only ESR was higher across calprotectin quartiles. Regarding cutaneous psoriasis, the severity of skin involvement was significantly higher in the upper quartiles (*p* = 0.01). Similar results were obtained with subgroups of patients meeting the CASPAR criteria, or when excluding patients in remission according to the DAPSA score (DAPSA ≤ 4). Similarly, we observed the same association between calprotectin quartiles and skin involvement when focusing on patients with a disease duration of less than 24 months. In these different subgroups, we did not demonstrate any associations with joint involvement. In contrast to RA and axSpA, there was a higher percentage of females in the upper quartiles (*p* = 0.04).

**Table 4:** Characteristics of the patients with psoriatic arthritis stratified by calprotectin percentiles. All results are mean (with standard deviation), except those with an asterisk* which are median [with interquartile range] and those marked with a “^§^” which are number of patients (%). *Abbreviations*: *BMI* body mass index, *CRP* C-reactive protein, *ESR* erythrocyte sedimentation rate, *DAPSA* disease activity in psoriatic arthritis (< 15: low, 15–28: moderate, > 28 high disease activity), *DLQI* Dermatology Life Quality Index

Serum calprotectin quartile [μg/ml]	0–1.4	1.2–2.2	2.2–3.4	3.4–12	*p* value
*n*	60	59	59	59	
Disease duration, years	8.3 (8.7)	8.6 (8.3)	7.5 (8.7)	7.4 (7.9)	0.84
Age years	53.6 (12.5)	50.1 (11.1)	49.8 (12.8)	52.4 (12.4)	0.27
Gender = male^§^	37 (61.7)	39 (66.1)	34 (57.6)	26 (44.1)	0.04
BMI kg/m^2^	27.4 (4.9)	27.7 (5.2)	26.0 (4.6)	28.8 (6.7)	0.19
ESR mm/h*	6.0 [2.0, 12.0]	7.0 [2.0, 13.0]	8.0 [4.0, 12.0]	11.0 [6.0, 20.0]	0.01
CRP mg/l*	3.0 [1.0, 8.0]	2.5 [1.0, 8.0]	3.0 [1.0, 6.8]	3.4 [1.8, 7.8]	0.38
Tender joint count	3.9 (9.3)	3.3 (6.4)	4.1 (8.9)	4.8 (8.5)	0.84
Swollen joint count	1.8 (4.5)	1.3 (2.5)	1.1 (2.5)	2.2 (4.4)	0.33
DAPSA	18.2 (23.5)	15.6 (17.5)	18.4 (16.8)	21.1 (24.2)	0.80
DAPSA moderate/high^§1^	14/36 (38.9)	12/39 (30.8)	14/31 (45.2)	13/28 (46.4)	0.35
DAPSA remission^§2^	6/36 (16.7)	9/39 (23.1)	7/31 (22.6)	4/28 (14.3)	0.86
DLQI	3.2 (5.1)	3.0 (4.5)	4.5 (5.5)	4.6 (7.3)	0.44
Reported skin manifestation^§^	14/43 (32.6)	22/39 (56.4)	23/47 (48.9)	23/38 (60.5)	0.01

^1^Proportion of patients with an ASDAS score ≥ 2.1

^2^Proportion of patients with an ASDAS score < 1.3

## Discussion

This is the largest study of serum calprotectin in patients with RA, axSpA, and PsA including cross-sectional as well as prospective analyses, with supporting evidence of the role of calprotectin as a potential prognostic biomarker for joint-related rheumatic diseases. In our study, serum calprotectin levels in the HC group were similar to levels found in the literature, with a median level of 1.2 μg/ml. Consistent with previous reports, we found higher levels of serum calprotectin in RA, axSpA, and PsA patients compared to the healthy control group. In RA, serum calprotectin levels were associated with disease activity (SJC and CDAI) and disease severity (HAQ and Ratingen score). A significant association between calprotectin quartiles levels and articular inflammation on ultrasound PD was also observed. Despite the low sensitivity of calprotectin, it has a good specificity to differentiate RA patients from healthy controls (even in this established disease cohort where the majority of patients were treated and well controlled). In association to other biomarkers (mainly ACPA and RF), serum calprotectin may be helpful to detect inflammatory disease in patients complaining of arthralgia. However, further studies are required to confirm this hypothesis [9].

In axSpA, an association between serum calprotectin and physician global disease activity and ASDAS scores was found. Serum calprotectin does not seem to be a good marker for axial disease activity (BASDAI, BASFI). Nevertheless, it correlated more strongly with peripheral articular involvement, and a significant association between serum calprotectin quartiles levels and hip involvement was observed. To our knowledge, this is the first study showing an association of serum calprotectin level with large joint involvement in axSpA.

Surprisingly, we did not find any associations between disease activity and calprotectin levels in PsA patients. Other studies demonstrated a correlation between serum calprotectin and disease activity in a polyarticular PsA population [34] or in early PsA [26]. One explanation could be the low SJC in axSpA and PsA compared to RA patients (Table 1). Another explanation could be the distribution of joint involvement. As already mentioned, calprotectin levels were found to be higher in patients with polyarticular PsA [25]. In a previous work focusing on PsA subtypes in the SCQM cohort, Stekhoven et al. showed that only a minority of patients had a polyarticular pattern (16 out of 957) in this Swiss cohort [35]. Finally, an interesting study observed a different distribution of calprotectin expression in the synovial tissue of patients with PsA compared to RA and SpA [11]. This might also suggest a different role of calprotectin in the physiopathology in PsA.

The serum calprotectin levels are influenced by local inflammation and synovial fluid calprotectin levels [10, 36]. Thus, we hypothesize that serum calprotectin levels reflect the amount of synovial inflammation, which is higher in polyarticular disease or in large joint involvement such as the hip. This observation is further supported by a correlation between calprotectin and the USPD score, with calprotectin outperforming CRP. The swollen joint count was also significantly different across calprotectin quartiles, even in the subgroup of patients with normal CRP levels. Recently, a prospective study of RA patients with normal CRP levels confirmed an association between calprotectin levels and disease activity in multivariate analyses [37]. Similarly, in a cross-sectional study of 87 patients receiving a TNF inhibitor, calprotectin seemed to be more accurate than the acute-phase reactants to discriminate disease activity [38].

Our results also suggest that calprotectin could be a useful biomarker for monitoring disease activity in RA patients and may add additional information to that provided by CRP. This observation is further underlined by the superiority of calprotectin over CRP in patients treated with tocilizumab. Indeed, as opposed to CRP, calprotectin is not produced by hepatocytes in response to inflammatory cytokines. It is mainly released via passive mechanisms when tissues are damaged (through necrotic cells and formation of neutrophil extra-cellular trap). Calprotectin is also a good marker of neutrophil activation and can therefore be more useful than CRP in some diseases, like RA, where neutrophil activation is present [39]. Therefore, calprotectin is not considered as a bona fide acute phase protein and could represent a good alternative when determination of acute-phase proteins cannot be reliably used such as in patients on anti-IL-6 therapies.

Interestingly, higher calprotectin levels were also associated with a higher prevalence of rheumatoid nodules, RF seropositivity, radiographic damage, and HAQ. These observations might suggest that serum calprotectin could also be a biomarker of disease severity and prognosis in RA, but this has to be further evaluated in a longitudinal study. Other authors have demonstrated that calprotectin is associated with radiographic progression [14] and cardiovascular risk [40]. Biologically, it has been shown that the principal extracellular effect of calprotectin is to amplify the inflammatory process through several mechanisms which may contribute to joint damage. Acting as a DAMP, it facilitates the activation of the TLR-4 signaling [41, 42], leading to secretion of proinflammatory cytokines such as IL-1, IL-6, and TNF, via the activation of NF-kB and p38 MPAK pathways [43].

This study has several limitations. Firstly, no longitudinal samples were collected, and consequently, we were unable to study the evolution of the calprotectin levels over time. Secondly, most of the patients had long-standing disease, and practically, all patients were on treatment with 40.8 to 57.5% of participants on a biologic, agent depending on the diagnosis. This may reduce the probability of demonstrating a positive association with the serum calprotectin level. Nonetheless, we demonstrated several significant associations between calprotectin and disease activity in both RA and axSpA. Despite the availability of detailed clinical characteristics, some characteristics such as the PASI skin score were not available. Another potential limitation is that the blood samples were not always performed on the same day as the clinical evaluation, although the time difference was minimal and unlikely to have influenced the results.

The strengths of this study include the large cohort size that is representative of the general population with inflammatory joint disease, including patients from outside tertiary hospitals, as well as the availability of detailed clinical, biological, and imaging data.

## Conclusions

This large study provides further evidence for the role of serum calprotectin as a potential biomarker for monitoring disease activity in RA patients. Furthermore, calprotectin represents a useful biomarker when CRP is normal or difficult to interpret, such as in patients treated with medications that suppress IL-6. We also observed an association between the serum calprotectin level and disease activity of axSpA patients, especially those with hip involvement. Conversely, in our oligoarticular-predominant population of PsA patients, we did not observe any associations between serum calprotectin levels and disease activity.

## Supplementary information

**Additional file 1 : Figure S1**. Receiver operating characteristic (ROC) curve of serum calprotectin as a disease activity marker in early rheumatoid arthritis. **Figure S2**. Ultrasound score in Rheumatoid Arthritis (RA): Calprotectin versus C-reactive protein (CRP). **Figure S3**. Swollen Joint Count (SJC) in rheumatoid arthritis patients treated with Tocilizumab: calprotectin versus C-reactive protein. **Figure S4**. Disease Activity Score in rheumatoid arthritis patients on tocilizumab: Calprotectin versus C-reactive protein (CRP). **Figure S5**. Ratingen score evolution over time for each calprotectin quartile levels in the RA population.

## Data Availability

All data generated or analyzed during this study are included in this published article and its supplementary information files.

## Abbreviations

ACPAs: Anti-citrullinated peptide antibodies
ASAS: Assessment of Spondyloarthritis International Society
ASDAS: Ankylosing Spondylitis Disease Activity Score
AxSpA: Axial spondyloarthritis
BASDAI: Bath Ankylosing Spondylitis Disease Activity Index
BASFI: Bath Ankylosing Spondylitis Functional Index
CASPAR: Classification Criteria for Psoriatic Arthritis
CDAI: Clinical Disease Activity Index
CRP: C-reactive protein
DAPSA: Disease Activity index for PSoriatic Arthritis
DAS: Disease Activity Score
DLQI: Dermatology Life Quality Index
ESR: Erythrocyte sedimentation rate
FDRs: First-degree relatives
GS: Grayscale
HAQ: Health Assessment questionnaire
HC: Healthy controls
IRD: Inflammatory rheumatic diseases
PASI: Psoriasis Area and Severity Index
PD: Power Doppler
PRO: Patient-reported outcome
PsA: Psoriatic arthritis
RA: Rheumatoid arthritis
RADAI: Rheumatoid Arthritis Disease Activity Index
RF: Rheumatoid factor
ROC: Receiver operating characteristic
SCQM: Swiss Clinical Quality Management registry
SJC: Swollen joint count
TJC: Tender joint count
USPD: Ultrasound power Doppler
