# Supplementary material for: Serum calprotectin: a promising biomarker in rheumatoid arthritis and axial spondyloarthritis
Source: Arthritis Res Ther. 2020 May 6;22:105. doi: 10.1186/s13075-020-02190-3 (PMC7201559; doi:10.1186/s13075-020-02190-3)

**Supplementary materiel (figures)**

***Figure S1: Receiver operating characteristic (ROC) curve of serum calprotectin as a disease activity marker in early rheumatoid arthritis.*** *Moderate/high activity (n=58) versus low activity (n=53), according to DAS28 ESR. Thresholds of 3.5* *µg/ml and 2.5 µg/ml (threshold with the highest sum sensitivity + specificity) and confidence interval for sensitivity are plotted. Area under the curve: 0.74. Boxplots of median calprotectin levels (with interquartile range) of the two groups are also shown (Wilcoxon test was used to compare median levels). Abbreviations: Sn, sensitivity; Sp, specificity*

*
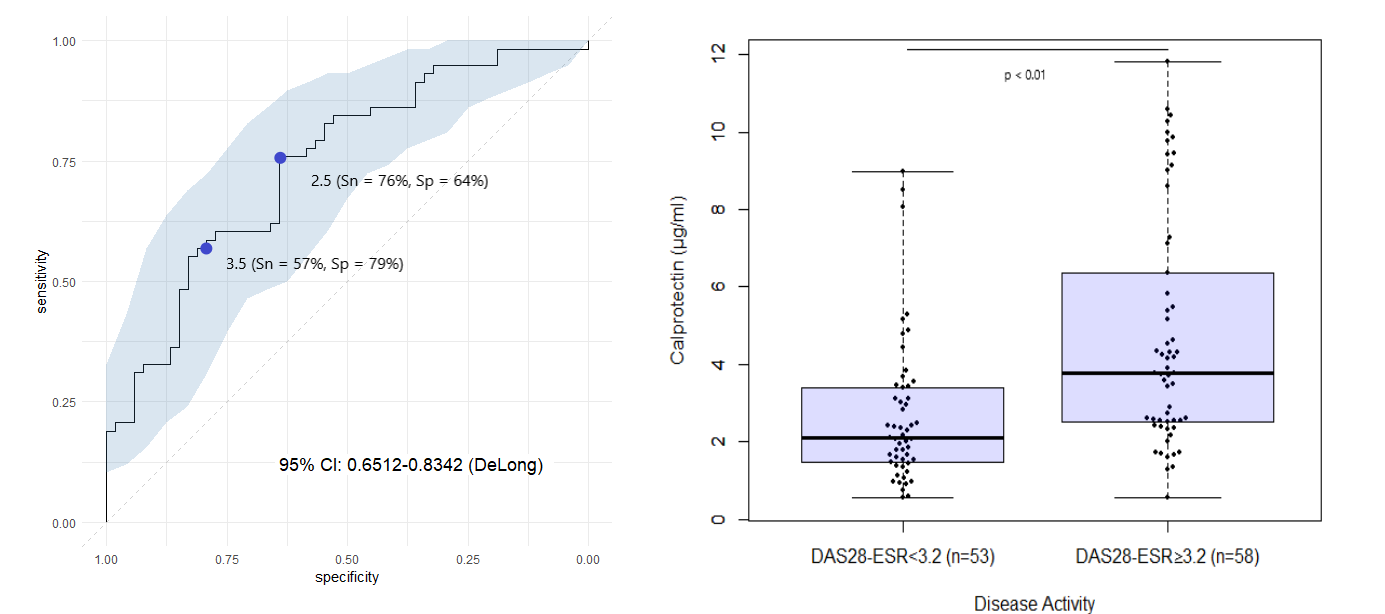
*

***Figure S2****:* ***Ultrasound score in Rheumatoid Arthritis (RA): Calprotectin versus C-reactive protein (CRP).*** *Receiver operating characteristic (ROC) curve of the ability of serum calprotectin and CRP to detect a positive Swiss Sonography in Arthritis and Rheumatism (SONAR)* *score in patients with RA. For calprotectin, thresholds of 1.5 µg/ml (mean serum calprotectin in HC), 3.5 µg/ml and threshold with the highest sum sensitivity + specificity are plotted. For CRP, thresholds of 5.0 mg/l (usual threshold) and threshold with the highest sum sensitivity + specificity are plotted. Abbreviations: AUC, Area Under Curve, 95%CI, 95% Confidence Interval; Sn, Sensitivity; Sp, Specificity.*

***A:*** *ROC curves on the 209 patients who had an ultrasonography examination: 147 had a positive SONAR score, and 62 were negative.*

***B:*** *ROC curves on 82 patients with ultrasonography examination and a disease duration less than 5 years: 55 had a positive SONAR score, and 27 were negative.*

**C:** *Boxplots of median levels (with interquartile range) of calprotectin and CRP in the 209 RA patients with SONAR negative score versus SONAR positive* *(Wilcoxon test was used to compare median levels).*


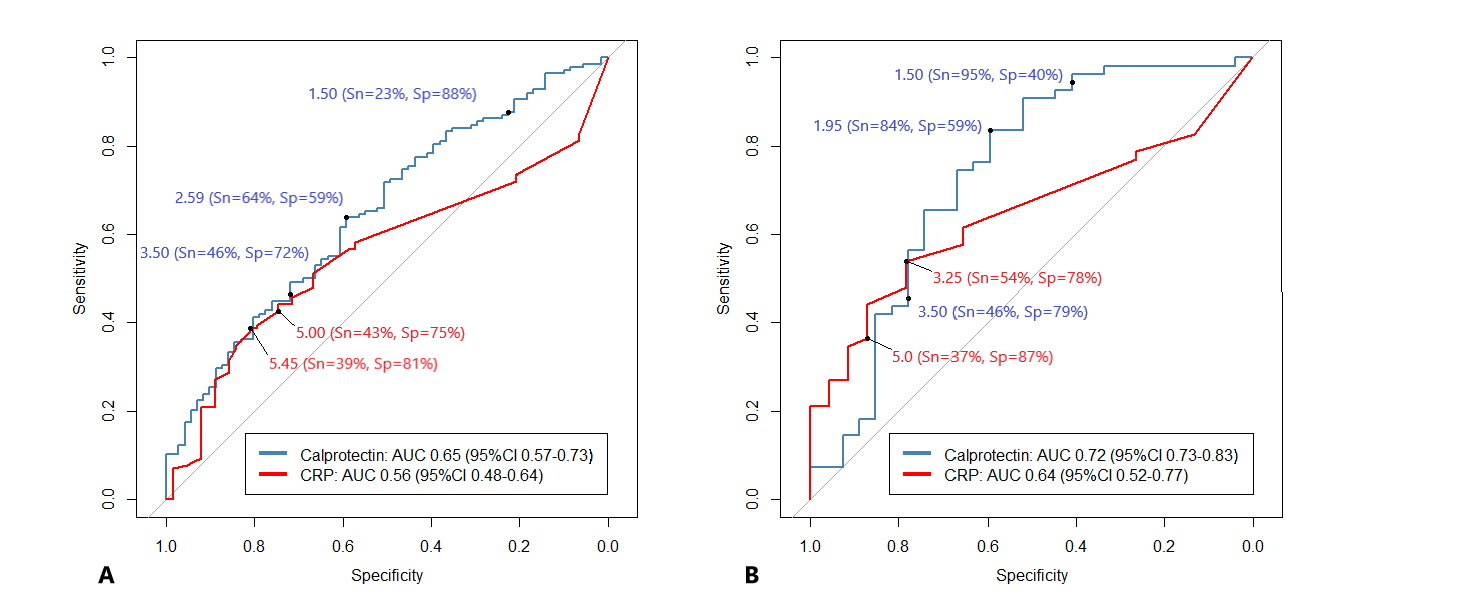


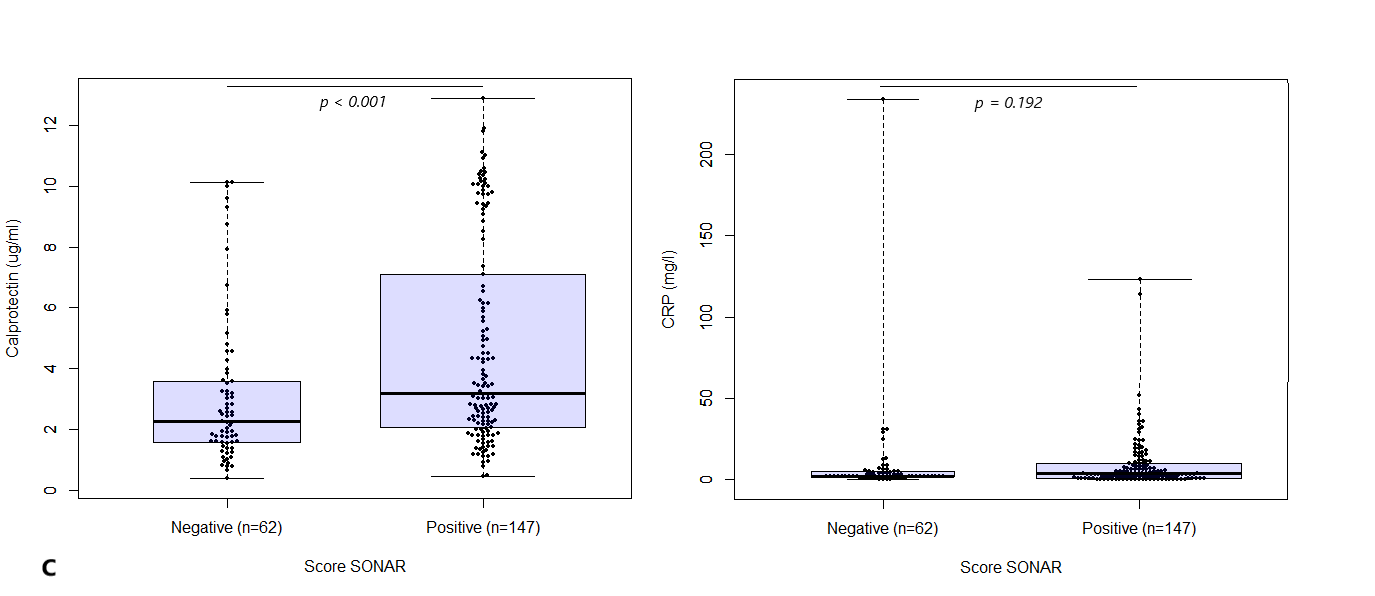


***Figure S3:*** ***Swollen Joint Count (SJC) in rheumatoid arthritis patients treated with Tocilizumab: calprotectin versus C-reactive protein.***

*Boxplots of median levels (with interquartile range) of calprotectin (A) and C-Reactive Protein (CRP) (B) in 51 RA patients treated with tocilizumab without swollen joint versus one or more swollen joint. Wilcoxon test was used to compare median levels.*


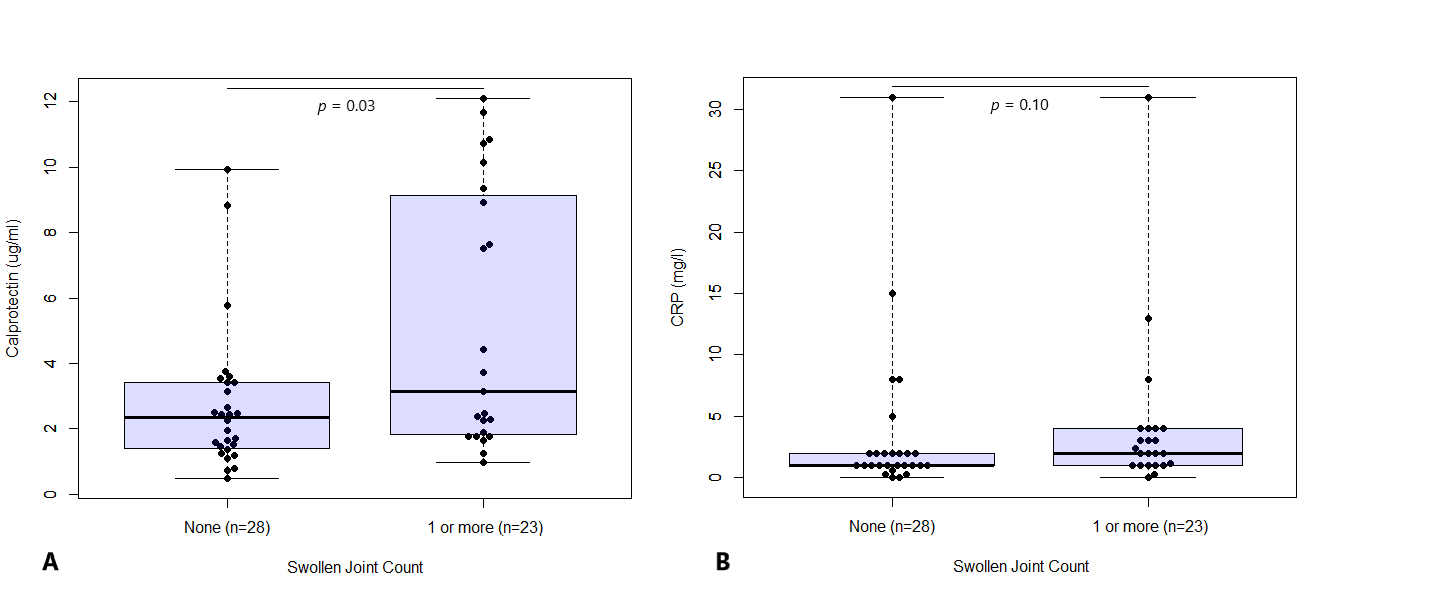


***Figure S4:*** ***Disease Activity Score in rheumatoid arthritis patients on tocilizumab: Calprotectin versus C-reactive protein (CRP)***

***A.*** *Boxplots of median levels (with interquartile range) of calprotectin and CRP in RA patients treated with tocilizumab with low disease (DAS28-ESR < 3.2) versus moderate to high disease activity (DAS28-ESR ≥ 3.2). Wilcoxon test was used to compare median levels.*

***B.*** *Receiver operating characteristic (ROC) curve of the ability of serum calprotectin and CRP to detect a DAS28-ESR above 3.2 in the same population. For calprotectin, thresholds of 3.5 µg/ml and 3.735 µg/ml (threshold with the highest sum sensitivity + specificity) are plotted. Area under the curve: 0.80. 95% CI: 0.66-0.96 (DeLong). For CRP, thresholds of 5.0 mg/l (usual threshold) and 2.7 µg/ml (threshold with the highest sum sensitivity + specificity) are plotted. Area under the curve: 0.71. 95% CI: 0.56-0.91 (DeLong)*


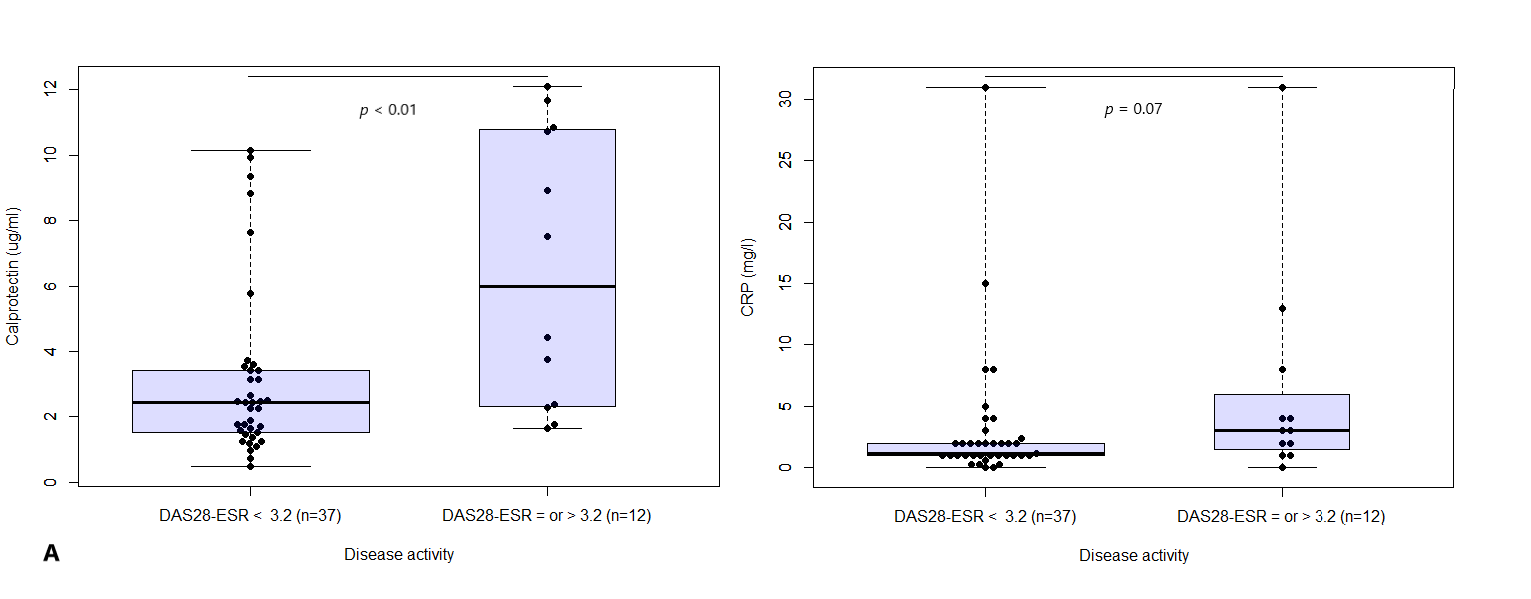


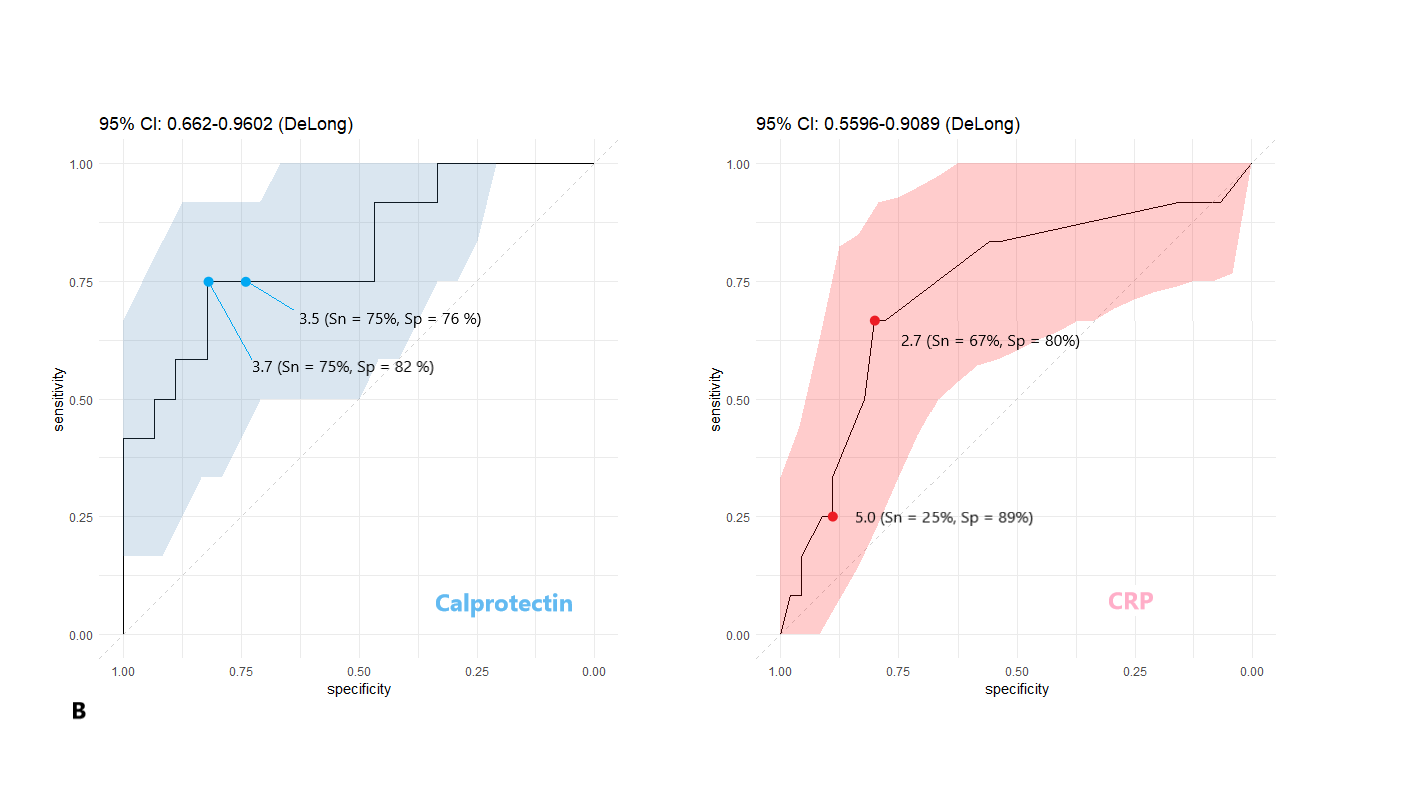


***Figure S5:*** ***Ratingen score evolution over time for each calprotectin quartile levels in the RA population.***


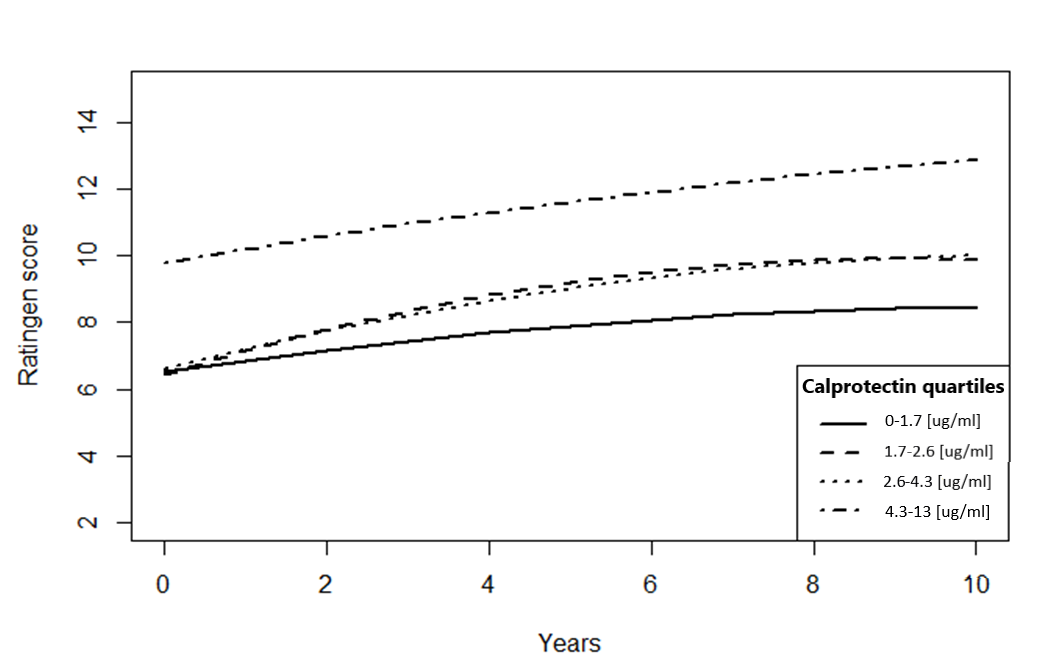

Supplement: Supplementary file 1 — Additional file 1 : Figure S1. Receiver operating characteristic (ROC) curve of serum calprotectin as a disease activity marker in early rheumatoid arthritis. Figure S2. Ultrasound score in Rheumatoid Arthritis (RA): Calprotectin versus C-reactive protein (CRP). Figure S3. Swollen Joint Count (SJC) in rheumatoid arthritis patients treated with Tocilizumab: calprotectin versus C-reactive protein. Figure S4. Disease Activity Score in rheumatoid arthritis patients on tocilizumab: Calprotectin versus C-reactive protein (CRP). Figure S5. Ratingen score evolution over time for each calprotectin quartile levels in the RA population. [file 13075_2020_2190_MOESM1_ESM.docx]
